# Sclerosis in Scattered Patches

**Published:** 1877-04

**Authors:** 

**Affiliations:** Paris


### Selections.

### SCLEROSIS IN SCATTERED PATCHES. BY PROF. CHARCOT, PARIS. TRANSLATED BY THOMAS OLIVER, M. B., PRESTON, WITH M. CHARCOT'S PERMISSION.

PART III. *Apoplectiform Attacks in Sclerosis in Patches — Periods and Forms — Physiologico-Pathology — Ætiology—Treatment.*

I purpose to-day to call your attention, in the first place, to certain cerebral accidents which may arise and complicate the symptomatology of cerebro-spinal sclerosis in patches. The accidents in question are *apoplectiform attacks*, which sometimes occur repeatedly, and which occasionally terminate fatally. These attacks have not as yet occurred in Miss V., whose clinical history is otherwise so complete in many other respects; but nothing justifies me in affirming that they will not happen some day. Indeed, it is not a rare complication: I find it pointed out in about a fifth of the cases that I have collected, and I myself have, personally, observed it at least in three.

The aggregate of symptoms which characterize the attacks in question do not belong properly to multilocular sclerosis. They present themselves in a number of diseases which affect at once several points of the cerebro-spinal axis—in particular, progressive general paralysis. It is even in this last disease that *congestive attacks* (this name usually serves to designate them, at least in France) have been particularly studied in proportion to their frequency. In these attacks we meet them under the rather various forms which they may assume. Accordingly, the description of these attacks in progressive general paralysis has occasioned numerous divisions and subdivisions. But, in short, all the varieties of form which clinical observation has brought into notice (and here I only wish to consider attacks of some intensity) may be reduced, if I am not mistaken, to two fundamental types, that is—1*st*, *Apoplectiform attacks* (*pseudo-apoplexy* of English physicians);

2nd, *Convulsive* or *epileptiform attacks.* The characters of the two types may also be intermixed and confounded in the same attack. Only the first type has been met with, until now, in sclerosis in patches; but there is no doubt that observations relative to this affection, by being repeated, will one day enable us to complete the picture.

Among the other organic diseases of the nervous centres in which we frequently observe epileptiform or apoplectiform attacks, I shall confine myself to particularize certain old *focalized* cerebral lesions, accompanied by permanent hemiplegia. Such are *cerebral hemorrhage* and *softening of the brain*, when they have occupied regions of the encephalon, the lesion of which has for its immediate consequence an almost certain determination of cerebro-spinal affections, known under the name of *descending fasciculated sclerosis.*

Between these partial lesions of the brain and progressive general paralysis it seeems, at first sight, as if there existed no point of contact. Here is, however, a character which brings them into relationship; the observations of M. Magnan and those of M. Westphal have shown that, in general paralysis, there is very often superadded to the lesions of periencephalitis a sclerotic degeneration, sometimes diffused, at other times fasciculated, which occupies at once the cerebral peduncles, the protuberance, the bulb, and certain regions of the spinal cord. Now, these cerebro-spinal lesions, as much from their mode of distribution as by the very nature of the morbid process, deserve to be assimilated to the descending fasciculated sclerosis consequent upon hemorrhage or softening of the brain. We know, on the other hand, that, in multilocular sclerosis, the sclerous patches occupy not only the spinal cord and the brain properly so-called, but, moreover, very generally various parts of the isthmus of the brain, and, in particular, the bulb. You see from this that the existence of irritative lesions scattered slightly everywhere in the cerebro-spinal axis, but always present in the isthmus, is a character common to all the affections, so dissimilar in appearance, to which are superadded the attacks called *congestive.* I shall draw your attention specially to the constant existence of bulbar lesion, which, in all likelihood, is a predominant element in the production of these attacks.

Be that as it may, the case before us is one of permanent degeneration of a slow and progressive development. It consequently cannot explain, without the concurrence of other lesions, the development of accidents which are produced, as a rule, almost suddenly, and which may disappear very quickly without leaving any trace. I am aware that many physicians, even at the present day, represent as supervening here a partial sanguineous congestion, a fluxion which, according to the necessities of the cause, would affect such and such a part of the brain; I cannot, for my own part, subscribe to this hypothesis. To justify my scepticism in this regard, I would at first appeal to the memory of those among you who are attached to the service of the lunatic asylum. How many times have they not been disappointed in not meeting at the autopsy the congestive lesion on which they had reckoned? But I will appeal particularly to the observations which I have been able to collect in the usual sphere of my labors. Many a time I have seen patients die in consequence of attacks, either epileptiform or apoplectiform, who had been affected for a long time with hemiplegia from the fact of softening of the brain or intracranial hemorrhage. Well, in such a case, whatever attention I paid to the autopsy, it has always been impossible for me to discover, either in the nervous centres or in the viscera, a recent congestive or œdematous lesion, or any other capable of explaining the grave symptoms which had marked the fatal termination: I have never met with anything but old lesions—ochreous foci, yellow patches or centres of cellular infiltration— on which depended the hemiplegia and the secondary degenerations of the mesencephalon and of the cord, which are the consequences of these partial lesions of the hemispheres. I believe, in short, that in the actual state of science the absence of proper lesions is, anatomically speaking, a character common to those attacks, whatever may otherwise be the form which they affect and the disease with which they are connected.

As regards the symptomatology of the apoplectiform and epileptiform attacks, (in order not to enter into the details of a regular description) I shall limit myself to the mention of the following particulars. The scene, in general, opens unexpectedly, without very marked antecedent symptoms; sometimes by a rapid and more or less distinct clouding of the intellectual faculties, and sometimes by deep coma supervening suddenly. There are added to this, in certain cases, convulsions which remind you of those of ordinary epilepsy, but which are nevertheless localized, as a rule, to one side of the body (*epileptiform attacks*). At other times, the convulsions are wanting (*apoplectiform attacks*). In both cases we frequently see developed from the beginning a more or less complete hemiplegia, sometimes with flaccidity, sometimes, but more rarely, with rigidity of the paralyzed limbs. The symptoms may abate progressively in the space of some days, and yet lead to death. This is announced in general by the rapid development of eschars in the sacral region. If, on the contrary, the patient is to survive, the disappearance of the accidental symptoms is not long in ensuing; the hemiplegia is the only symptom which persists still for a time, but it disappears, sooner or later, without leaving any trace.

The attacks are usually reproduced several times, generally after long intervals, during the course of the disease. As far as regards sclerosis in patches, they have been noted three times in the observation III. in the memoir of M. Vulpian, three times in the reeorded observations of Zenker, and even seven times in that of M. Leo. These fits have always left behind them a notable and persistent aggravation of all the symptoms of the primary disease.

The sketch which I have just presented to you would be too imperfect, if I did not draw your attention to the disturbances of the circulation and of the temperature which, as a general rule, are manifested in the course of the attacks. The *pulse* is found always more or less accelerated; but, moreover (and

---

[1] A temperature of 38° C. is subfebrile, and is equivalent to 100.4° F. A moderately febrile state would be indicated by a temperature of 39° C. (102.2° F.). 40° C. (104° F.) indicates a highly febrile condition. Temperatures above this level are hyperpyretic, and are usually dangerous to life, *e.g.*, 42° C. (107.6° F.).

this is the important point), the *temperature* of the central parts rises rapidly: it may, in the short time closely subsequent to the invasion, reach 38° C., or even 39° C[1]. Frequently, at the end of twelve or twenty-four hours, it rises up to 40° C., and maintains itself at this figure for some hours without any material alteration in the condition of the patient. But if the patient is to survive, the temperature soon rapidly decreases. A figure above 40° C. almost always brings on the fatal termination.

These modifications of the central temperature have been studied by M. Westphal in the epileptiform and apoplectiform attacks of *progressive general paralysis:* I have found them in the attacks which supervene in the case of patients affected by old hemiplegia consequent upon hæmorrhage, or softening of the brain. In order to give you more definite ideas upon this subject, I think it useful to present to you very briefly the details of two observations relating to cases of the last kind.

The first observation has reference to a woman aged thirty-two years, affected with a hemiplegia of the right side, dating from infancy. There was general atrophy, rigidity, and shortening of the limbs, and paralysis, as is generally seen in such cases. This woman was subject to epileptiform attacks. She was brought to the Infirmary some hours after the beginning of an attack more intense than usual. On the very evening of her admission the temperature was beyond 38° C., and on the following day it had reached 40° C. The fits became subintrant: they were repeated about a hundred times a day. Eschars rapidly formed in the sacral region, and death supervened on the sixth day. The thermometer introduced into the rectum stood that day at 42.4° C. At the autopsy we found on the surface of the cerebral hemisphere of the left side a considerable depression, corresponding to a yellow patch, the vestige of a vast focus of softening. The hemisphere was besides atrophied throughout. We were unable to discover any trace of a recent lesion either in the nerve-centres or in the viscera.

The second case is that of a woman of sixty-one years of age, affected with right hemiplegia, consequent upon a cerebral hæmorrhage of two years' date. This woman had already experienced several epileptiform or apoplectiform attacks, otherwise *generally rather slight.* Two hours after the commencement of the symptoms, the temperature taken in the rectum was 38.8 ° C.—five hours later it rose to 40 ° C. On the following day, in spite of the cessation of the convulsions, the temperature was 41 ° C., and on the day after that (the day of death) it reached 42.5 ° C. Two ochreous masses were discovered at the autopsy, the one seated in the corpus striatum, and the other in the substance of one convolution There existed no recent lesion capable of explaining the symptoms which had determined death.

It has not yet been granted me to follow day by day, and at various periods of the day, the evolution of the temperature in a case of *apoplectiform attack* supervening in a patient affected with *sclerosis in patches.* Nevertheless, we can gather from several observations partial results, which leave no doubt that, even under this point of view, things proceed in multilocular sclerosis exactly as in progressive general paralysis, and in cases of focalized lesions of the hemispheres. Thus the patient, whose history has been reported by Zenker, was seized towards the end of his life by an apoplectiform attack, followed by hemiplegia of the right side of the body. On the day of the attack, the pulse being 136, the temperature reached 39·6 ° C. On the following day the thermometer marked 40 ° C., and on the day after this the paralysis had improved, and the temperature again fell to the physiological standard. In the patient Nolle, recorded by M. Leo, an apoplectiform attack declared itself during the evening. Early on the following morning the pulse was 144, and the temperature was 38.5 ° C. This attack, the seventh which the patient had experienced, was destined to terminate in death during the night. In the case of N., whose history has been recorded, in my service, by M. Joffroy, five hours after the invasion of an apoplectiform attack, with incomplete loss of consciousness and general relaxation of the limbs, the rectal temperature was 40·3 ° C., and the pulse 120. On the following day the apoplectiform symptoms had disappeared, and, at the same time, the pulse as well as the temperature had returned to the normal state.

If I have dwelt at some length upon the modifications which the temperature undergoes, in the apoplectiform and epileptiform attacks of general paralysis, and of some other cerebro-spinal affections, it is because, in my opinion, we find in them a character which can, in certain cases, be turned to profit in the diagnosis. It is not necessary, I think, to enter into long details to show how difficult it is in presence of a patient who has just been struck with apoplexy, with or without accompanying convulsions, to decide in accordance with the mere consideration of external symptoms, whether it is a case of *true apoplexy*, resulting from the actual formation of a cerebral clot from hæmorrhage or from softening, or, on the contrary, from a simple *congestive attack*. Now, the examination of the temperature of the internal parts would furnish in such an occurrence a fact decisive of the case. Indeed, I have demonstrated by repeated observations that in true apoplexy, principally when it is connected with cerebral hæmorrhage, the temperature constantly falls soon after the attack, and is maintained, generally during twenty-four hours at least, below the normal rate, even when there ensue and are repeated intense convulsive fits. Now, we have just seen that in the attacks called congestive, the temperature, on the contrary, rises from the commencement of the first symptoms beyond the physiological figure, and tends still to rise progressively during the fit.

### *Periods and Forms in Sclerosis in Patches.*

After having considered one by one the various elements which compose the symptomatology of multilocular sclerosis when there is question of a complete case, and one which has already reached an advanced period of its course, it is expedient to show by a general survey how these elements are grouped together and connected with the various phases in the different forms of the disease. This latter does not present itself actually—far from it—clad in all its attributes at every epoch of its development. At the commencement, it may be constituted only by the union of two or three symptoms, and, besides, there are cases in which up to the fatal termination, the delineation of the symptoms remains incomplete. Now, it is especially when the disease is still at a period near its commencement, or when it takes on an imperfect form, that it would be of consequence to be able to recognize it by the smallest indication.

I have proposed, in the progressive development of the malady, three periods: the first extends from the moment at which the earliest symptoms appear, up to the time when spasmodic rigidity of the limbs reduces the patient to an almost absolute impotence. The second comprises all the time (generally very long) during which the patient, confined to bed, or hardly able to take a few steps in his room, preserves, nevertheless the integrity of his organic functions. The third, in fine, commences at the moment when, at the same time as all the symptoms of the disease are aggravated simultaneously, the functions of nutrition suffer in a perceptible manner. *Apropos* of this last period, there will be occasion to notice the accidents which, in the usual order of things, mark the last period of the disease, and precipitate the fatal termination.

I. First Period.—The mode of invasion and the linking of symptoms present various aspects, which deserve to be pointed out to your special attention.

Sometimes it is cephalic symptoms which open the scene: thus, the patients begin by complaining of habitual vertigo, of diplopia more or less transient; gradually embarrassment of speech appears, and finally, nystagmus. The union of these symptoms would compose a group already characteristic enough, and one, which, even when the trembling caused by movements and the paresis of the limbs do not come sooner or later to be united with it, would yet allow of the diagnosis being established upon strong presumptions.

But such is not the commonest mode of invasion; most frequently it is the spinal phenomena which first appear; so much so, that during several months, and sometimes even several years, the patients may present no other symptoms than a weakening, a paresia, more or less marked, of the lower limbs, tending to become aggravated in a slowly progressive manner, and to be extended to the upper limbs. In such a case, the situation of the clinical observer is necessarily one of the most difficult. For, to sum up, the paresia of the lower limbs is a very ordinary symptom, and one common to a number of various affections. It presents itself, however, in multilocular sclerosis, as you know, with some peculiar traits, which might perhaps point out the way. Thus, however marked it may be (setting aside the exceptional case in which the lesion would predominate in the posterior columns,) it is not accompanied by any disturbance of the sensibility, nor any appreciable change in the nutrition of the muscular masses; besides, there is ordinarily connected with it no functional disorder in the bladder or the rectum; in fine, it is not rare to see remissions produced, nay, even complete intermissions which have afforded grounds to hope for an ultimate cure. But it is clear that these indications, even with the concurrence of all the others, can only furnish information of rather a vague description. Certainty can hardly be attained, except where the special trembling, or some one of the cephalic symptoms, comes to be superadded to the spinal symptoms.

Thus far have I presented to you the invasion and the ulterior concatenation of accidents as slow and uniformly progressive. That, in effect, is by far the most frequent case; but it is important that you should know that, in certain circumstances (exceptional, it is true), the commencement may appear suddenly, unexpectedly, or be consequent upon some comparatively insignificant premonitory symptoms.

Thus the vertigo and diplopia having suddenly declared themselves, the paresia of the limbs and the stumbling may have been superadded to them at the expiration of some days, so that the malady is found, so to speak, at once established. This is what took place, among others, in the case of a young patient, Mlle. Vinch, whom some of you may have seen in our wards. At other times the beginning is marked, as in one of the patients of Valentiner, by a sudden invasion of paresia in one of the lower limbs; or, again, as in the case of M. Leo, and in one of my patients, whose history M. Vulpian has reported, an apoplectform attack, preceded for several days or several weeks by vertigo and cephalalgia, and followed by temporary hemiplegia, inaugurates the illness.

Lastly, there is a case to which I will again call your attention, and in which the beginning is marked by an affection which is most frequently considered accidental and extraneous to the leading malady, though, in my opinion, on the contrary, it is really connected with it by a link not recognized till now I allude to *gastric* or *gastralgic crises* (whichever you may choose to call them), which are sometimes intense, and accompanied by lypothemia, repeated vomiting, etc. They have several times opened the scene, and soon the usual symptoms of multilocular sclerosis have followed; besides, it is not unusual to see them re-appear at several successive periods, and be intermingled with the symptoms during the first periods of the disease. In this class, a case published by M. Liouville, and that reported by M. Zenker, are good examples to quote. These accidents are so much the more worthy of notice, as we shall find them again with almost the same characters in other forms of sclerosis of the spinal cord, and, in particular, in posterior fasciculated sclerosis (*locomotor ataxy*), chiefly, however, in the initial phase of this affection. The gastric crises, coinciding, or alternating with sudden flashing pains of the limbs, may be, in such a case, with diplopia, and perhaps a little titubation when the eyes are closed, the only actual symptoms of the disease in question, whose real character is at that time too often not recognized. These same gastric crises are met with, as we have observed (M. Duchenne, of Boulogne, and myself), in the form of *central subacute* or *chronic myelitis*, which produces the symptoms of *general spinal paralysis.* But I do not wish to dwell longer on this subject, which I expect to resume soon, and to give to it all the developments of which it is susceptible.

II. Second Period.—In general, from the end of the first period, multilocular sclerosis presents itself already marked by most of the symptoms which characterize it. These symptoms are aggravated and intensified during the second period, and there is superadded to them the spasmodic contraction of the limbs, with or without the accompanying spinal epilepsy, in consequence of which the patients, who had till then been still able to walk more or less effectually unaided, find themselves thereafter reduced to an almost absolute impotence, and confined ultimately to their room, or even their bed. The contraction which characterizes the commencement of this period is a phenomenon almost always slowly developed; it hardly ever shows itself, in general, until two, four, or six years after the appearance of the first symptoms of mutilocular sclerosis.

III. Third Period.—The commencement of this last period is marked, as I have told you, by progressive weakening of the organic functions; want of appetite is habitual, and diarrhœa frequent, and soon there supervenes a general emaciation, which becomes more and more evident. At the same time, there is an aggravation of all the symptoms peculiar to the disease: the clouding of the intellect proceeds even to imbecility; the embarrassment of speech is carried to its height, and the patient no longer expresses himself except by an unintelligible grunt. Then the sphincters are paralyzed, and it is not unusual to see the mucous membrane of the bladder become the seat of an ulcerative inflammation. It is then that appear in the sacral region and on all points of the lower limbs submitted to prolonged pressure, eschars, which sometimes assume enormous proportions, and consecutively all the series of accidents which are connected with this complication, such as purulent inflammatory foci, purulent or putrid poisoning, etc. Death soon ensues.

Most frequently life is still abridged by the intervention of some intermittent malady. Pneumonia, caseous phthisis, and dysentery may be reckoned on as amongst the most frequent of these terminal affections.

I have reserved, so as to mention it in quite a special manner, the appearance of some symptoms of *bulbar paralysis*, because they may, by becoming aggravated, precipitate the course of events, and bring on a fatal termination, even before the phenomena of the last period have manifested themselves. At the same time, as the speech becomes more and more difficult, there is produced, in the first place, a difficulty of deglutition, which, transitory at first, soon becomes permanent. Then from time to time, there occur fits, more or less serious, of dyspnœa, and death may take place in one of these attacks. I have observed quite recently two cases which terminated in this way. At the autopsy, we recognized in both that a patch of sclerosis had invaded the floor of the fourth ventricle, where it united the points of origin of most of the bulbar nerves.

After the details into which I have just entered, it appears to me useless to undertake the particular description of the various *forms* that multilocular sclerosis may put on. The *cerebral* and *spinal* forms correspond to an incomplete invasion of the nerve centres by sclerosis; it is, if you like, the disease arrested in its development, in its progression, whether ascending or descending. The symptomalogical series is, so to speak, curtailed by it; but the symptoms, considered singly, are not on that account modified. The first form is very rare, the second frequent enough, on the contrary. The *cerebro-spinal* form, however, represents the normal type, and it is it we meet with most frequently in the clinique.

Cerebro-spinal multilocular sclerosis fully accomplishes its total development in the space of from six to ten years: that forms a new contrast with paralysis agitans, whose normal duration is much longer. The spinal form usually allows more respite; it may terminate only at the end of twenty years, or even at a still later period.

*Physiologico-Pathology.—Etiology: Prognosis and Treatment.*

To finish this study, it remains for me to speak of the physiologico-pathology, etiology, and treatment of multilocular sclerosis of the nerve-centres. Unfortunately, the documents which I shall be able to appeal to, relative to these various points, are few in number, and imperfect, too, for the most part. I shall be compelled in consequence to lay before you very summary remarks.

The reason of the very singular mode of distribution which the sclerous islets affect in the various parts of the central nervous system is to us till the present completely unknown. Rindfleisch has affirmed that the point of departure of the formation of sclerotic foci is in the vascular system. According to him, inflammation of the walls of the little vessels which we meet with always at the centre of the patches in course of formation is the initial fact: from this central part the irritation is propagated to the reticulum of the neuroglia, and radiates in all directions. Evidently that would be again only staving off the difficulty. Besides, this predominant part accorded to the vessels in the evolution of the morbid process is anything but demonstrated. I am very much disposed to think, in accordance with my own observations, that the degenerations of the vessels, and those of the recticulum, advance *pari passu* in a parallel manner, and without influencing each other.

Be that as it may, given the seat of sclerous islets in the various departments of the nerve-centres, can we deduce from that the production of symptoms of which the aggregate constitutes the symptomatology of sclerosis in patches? It is possible, at least in part. We have already remarked that the want of motor co-ordination, the loss of knowledge of position, and the flashing pains which are observed in a certain number of cases, may be, in these cases, referred to invasion of the posterior columns of the spinal cord. On the other hand, the habitual predominance of the patches of sclerosis on the passage of the antero-lateral columns, accounts, as I will demonstrate to you soon, for the almost constant existence of the paresia or paralysis of the limbs, followed sooner or later by permanent contraction. Nystagmus and embarrassment of speech are in connexion with the habitual localization of the patches in the substance of the protuberance and bulb. But a great number of other symptoms are more difficult of interpretation. Such is, amongst others, the peculiar tremor which shows itself in certain attitudes of the body, and in the exercise of voluntary movements. I have expressed the opinion that the protracted persistence of axis-cylinders, when deprived of their sheath of myelin in the midst of the sclerotic foci, plays perhaps an important part here: the transmission of voluntary impulses would still be effected by way of these denuded cylinders, but it would take place in an irregular spasmodic manner, and thus would be produced the oscillations which disturb the execution of intentional movements.

This resistance of the axis-cylinders is certainly not a phenomenon exclusively peculiar to multilocular induration, but it shows itself there more marked than in the other forms of sclerosis of the nerve-centres. It may, however, be appealed to, I think, for the slowness with which the paretic symptoms progress in sclerosis in patches, and for the long space of time that elapses before the period in which they give place to complete paralysis and permanent contraction.

What we know concerning the conditions which preside over the development of sclerosis in patches reduces itself to very little. It appears established, however, from the present, that the disease is much more common among women than among men. Thus, among the cases which I have collected in my early studies, three or four only concern men. The facts which have been published since then have not modified in any sensible manner this result. By combining with the eighteen cases which figure in the monograph of MM. Bourneville and Guérard, sixteen new cases, we have a total of thirty-four cases, of which nine were men and twenty-five women.

From these same documents, it results that it is a malady of youth, or of the first half of adult age. We have observed it in subjects aged from fourteen, fifteen, and seventeen years. But it appears most frequently to commence between the age of twenty and twenty-five. It rarely appears after thirty years. The age of forty years seems to be, on the other hand, the last limit that people affected with sclerosis in patches can attain.

With regard to hereditary influence, we would have to cite only a single example, in which it would appear to have played a certain part. This example has been communicated to us by M. Duchenne (of Boulogne).

In the pathological antecedents of the patients themselves, we have in general only very vague indications. Hysteria figures among them in some cases; but most frequently we find mentioned only neuropathic accidents of no marked description—migraine from time to time, or neuralgia.

Among the *occasional causes*, we find several times pointed out the prolonged action of moist cold. In one case, the first symptoms were developed soon after a fall.

But it is circumstances of a moral kind which are most commonly referred to by patients. Prolonged grief, for example, that, among others, which an illegitimate pregnancy may occasion; or, again, the unpleasantness and weariness which a social position more or less false entails, such as is often that of certain governesses. This is what concerns women. As for the men, the case before us is for the most part of unclassed people, placed outside of the general current, too susceptible and badly armed to sustain what we call, in Darwin's theory, the *struggle for life.* That is, in fact, a somewhat vulgar etiology, and which we find, so to speak, at the origin of all chronic diseases of the central nervous system.

The *prognosis* until now is of the gloomiest description. Will it always be the same? We hope that when the disease is better known, the physician will learn to take advantage of these spontaneous tendencies to remissions which are found prominently marked in a great number of cases. Besides, we must not forget that, for the present, the disease is in general only truly recognised when the lesions are already very profound, and nearly inaccessible to the influence of curative means.

After the preceding, shall I go on to speak to you at length on the therapeutics? The time is not yet come when this question can be seriously approached. I can speak to you only of a few trials made up till this day, and of which the results, unfortunately, have been in general not at all favorable.

*Chloride of gold* and phosphide of zinc appear to have rather exasperated the symptoms. *Strychnia* has sometimes made the trembling cease, but its influence has always been temporary. I shall say as much of the *nitrate of silver.* In several cases which I have observed, it appears to have had upon the trembling, and upon the paresis of the limbs, a very favorable influence, but one which, in truth, has not been long maintained. A formal contra indication to the employment of this medicine would be the existence of permanent contraction, and, above all, of spinal epilepsy. The employment of silver nitrate would, in fact, almost certainly have for its result exasperation of these symptoms. *Hydrotherapia*, in one case, seems to have produced a transient impression; in another, on the contrary, it has completely failed.

*Arsenic*, *belladonna*, *ergot of rye*, and *bromide of potassium*, have been equally administered in sclerosis in patches without any marked benefit. I will say as much for the application of *faradization*, and of the employment of the *continuous current*. But, with reference to this last agent, it is important to have recourse to new experiments before delivering a decided opinion on the qustion.—*Edinburgh Med. Journal, Oct. and Nov.*, 1876.

